# Endobronchial indocyanine green instillation to identify the intersegmental plane for successful segmentectomy

**DOI:** 10.1002/rcr2.1174

**Published:** 2023-06-19

**Authors:** Paul Lilburn, Jonathan Kwan, Jonathan Williamson, Kevin Ho‐Shon, Mohammad Azari, Michael Wilson, Alvin Ing, Tajalli Saghaie

**Affiliations:** ^1^ Department of Respiratory and Sleep Medicine Prince of Wales Hospital Sydney New South Wales Australia; ^2^ School of Health Sciences University of New South Wales Sydney New South Wales Australia; ^3^ Faculty of Medicine, Health and Human Sciences Macquarie University Macquarie Park New South Wales Australia

**Keywords:** bronch, Indocyanine green (ICG), intersegmental plane, pulmonology, Segmentectomy

## Abstract

The traditional indications for lobectomy for resectable Non‐small Cell Lung Cancer (NSCLC) may be set to change. Recently, anatomical segmentectomy (AS) versus lobectomy as an approach for early‐stage NSCLC has been described in phase 3 randomised controlled trials. The demand for methods to facilitate AS may increase as a consequence. We describe three cases of AS using the combination of endobronchial infiltration of indocyanine green (ICG) to identify the intersegmental plane (critical for the performance of AS), and Computed Tomography (CT) guided methylene blue injection for lesion localisation. The operations were completed successfully demonstrating satisfactory post‐operative outcomes including lesion resection with clear surgical margins and acceptable length of stay. We believe that endobronchial instillation of ICG and CT‐guided methylene blue injection for lesion localisation show promise as a technique to complement parenchymal sparing thoracic oncological surgery.

## Graphical abstract

We describe three cases of anatomical segmentectomy (AS) using the combination of endobronchial infiltration of indocyanine green (ICG) to identify the intersegmental plane (critical for the performance of AS), and Computed Tomography (CT) guided methylene blue injection for lesion localisation. The operations were completed successfully demonstrating satisfactory post‐operative outcomes including lesion resection with clear surgical margins and acceptable length of stay. We believe that endobronchial instillation of ICG and CT‐guided methylene blue injection for lesion localisation show promise as a technique to complement parenchymal sparing thoracic oncological surgery.

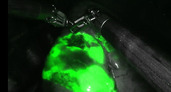

## INTRODUCTION

Lung cancer is the most common cancer diagnosis worldwide and results in over a quarter of all cancer deaths.^1^ It is estimated that lung cancer will contribute to 9% of all newly diagnosed cancer and 17% of cancer‐related death in 2022.^2^ The increased availability of computed tomography (CT) has led to the detection of earlier stage lung cancer.^3^, ^4^ The anticipated lung cancer screening programs will detect earlier stage disease which will be treated surgically.^3^

An increase in mortality and higher recurrence rates in patients treated with sublobar resections compared with lobectomy was noted in the 1990s.^5^ As a consequence, lobectomy had remained the gold standard surgical approach to date.

More recently, the surgical management for early‐stage NSCLC has been re‐examined. Overall survival was analogous to those ‘intentionally selected’ for sublobar resection versus lobectomy in one systematic review.^6^ Meta‐analyses assessing sublobar resection have found that anatomical segmentectomy (AS) produces a better overall survival rate, cancer‐specific survival, and disease‐free survival than wedge resection for stage 1 NSCLC.^7^, ^8^, ^9^ AS has also been associated with wider surgical margins, an increased number of sampled lymph nodes^10^ and an increased rate of nodal upstaging compared with wedge resection.^11^

The increase in survival from NSCLC has led to a rise in patients with metachronous tumours. These patients, who have already undergone a lobectomy, are often appropriate candidates for parenchymal sparing strategies. These factors have generated opportunities for more sophisticated, targeted approaches to parenchymal sparing surgical resection.

Of note, a phase 3 randomised controlled trial (RCT) published this year demonstrated AS compared with lobectomy in early‐stage non‐small cell lung cancers to have similar outcomes, promoting AS for consideration in this population.^12^

The intersegmental plane is critical for the accurate performance of AS.^13^, ^14^ Traditionally, this has been carried out using the inflation‐deflation method; however, limitations to this technique have been noted.^15^ Our paper describes the use of indocyanine green (ICG) as an endobronchial injection to identify the intersegmental plane in conjunction with CT‐guided injection of methylene blue to locate the target lesion and thus facilitate an accurate AS.

## CASE SERIES

### Patient A

A 74‐year‐old man with a history of childhood passive cigarette smoke exposure and paternal family history of leukaemia presented with a four‐month history of abdominal pain. Investigations revealed an incidental ground glass nodule measuring 7 × 6 × 12 mm abutting the horizontal fissure posteriorly in the right upper lobe. No distant metastatic disease or mediastinal/hilar lymph nodal involvement was detected on subsequent PET/CT imaging. Pre‐operative lung function testing demonstrated an FEV1 of 107% at 2.71L, FVC of 109%, normal volumes and normal DLCO.

A multidisciplinary decision was made to perform an AS. 1 mL of methylene blue was injected via CT guidance with a 22‐gauge spinal needle, followed by endoscopic ICG injection under flexible bronchoscopy. Blue dye was identified at RB2 consistent with a recent methylene blue injection. An Olympus B5‐2C balloon was placed and inflated inside RB2b to prevent spillage. 2.5 mLs of ICG was instilled into the segmental airway (RB2). Anatomical right upper lobe posterior segmentectomy was conducted under four‐port robotic surgery (Da Vinci system).

The patient was successfully discharged on day three post‐operation. Histopathology confirmed an 8 mm adenocarcinoma in situ with clear surgical margins. The three lymph nodes sampled were not involved. Stage TisN0 was confirmed.

### Patient B

An 84‐year‐old male smoker with a 30‐pack‐year history of exposure presented a two‐month non‐productive cough history. He had a past history of renal cell carcinoma treated with nephrectomy and prostate cancer for which he underwent combined brachytherapy and radiotherapy. A CT chest found a spiculated 12 × 12 mm lesion in the right upper lobe. PET/CT scan demonstrated no metastatic disease. CT‐guided biopsy confirmed lung adenocarcinoma with ERBB2 mutation.

Pre‐operative lung function testing demonstrated FEV1 of 106%, FVC of 138%, normal lung volumes and normal DLCO. The multidisciplinary decision was for AS. 10 mLs of ICG was instilled endobronchially via Olympus TH190 flexible bronchoscope into RB2, followed by 40 mLs of saline flush. The right upper lobe posterior segment was successfully resected robotically (Da Vinci system).

Post‐operatively, there was a small apical pneumothorax that settled on serial imaging. Histopathology confirmed mucinous adenocarcinoma with clear surgical margins. He was discharged on day five post‐operation. All five lymph nodes sampled were not involved. StageT1bN0 was confirmed. Post‐operative spirometry demonstrated unchanged values.

### Patient C

A 69‐year‐old man with a history of a left lower lobectomy in 2017 for stage I adenocarcinoma underwent routine surveillance CT chest, which identified a new 7 mm ground‐glass nodule in his right lower lobe in 2020. Subsequent PET/CT scan demonstrated no metastatic disease. His other significant background was a previous right nephrectomy for clear cell renal carcinoma.

**FIGURE 1:**
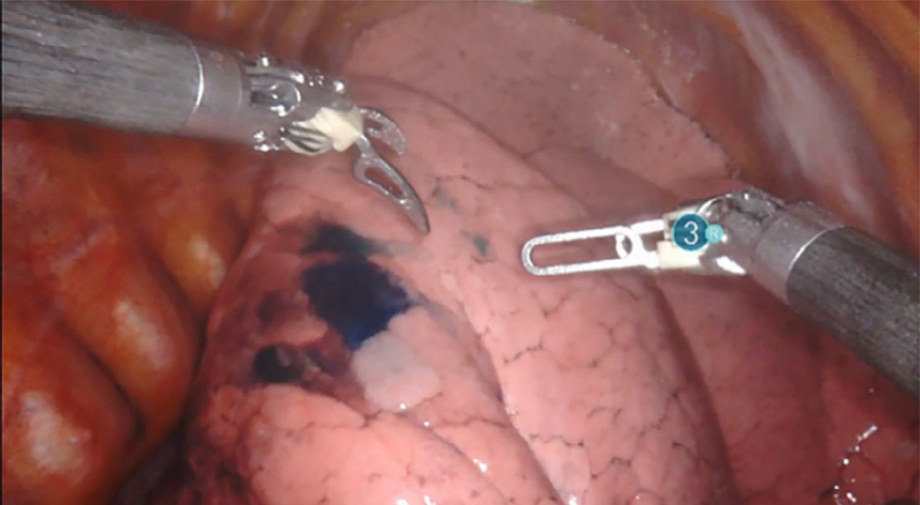
Methylene blue injected via CT‐guidance to right upper lobe lesion.

**FIGURE 2:**
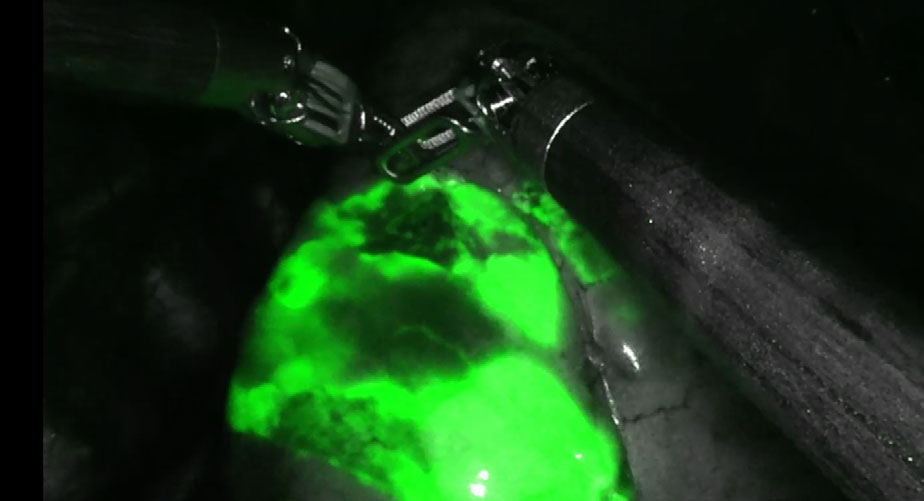
Immunofluorescence demonstrating the anatomical lung segment (RB6) visualized during robotic assisted thoracic surgery following endobronchial Indocyanine green (ICG) injection.

A multidisciplinary decision recommended AS. 1 mL of methylene blue was injected into the lesion under CT guidance with a 22‐gauge spinal needle (Figure 1), followed by endoscopic ICG instillation via flexible bronchoscopy using an Olympus Th190 bronchoscope. The target airway was identified (Figure 2) as the superior segment of the right lower lobe (RB6) and blocked with an Olympus B5‐2C balloon. 10 mLs of ICG was injected distally into the blocked airway, followed by 2 mLs of saline and 2m Ls of air. Successful robotic segmentectomy was conducted under four‐port VATS, with the superior segment of the right upper lobe resected.

Postoperatively he encountered a residual air leak managed with a chest drain. The patient was successfully discharged on day eight post‐operation after the pneumothorax had resolved.

Histopathology confirmed lung adenocarcinoma with clear margins. The two lymph nodes sampled were not involved, thus Stage T1bN0 was confirmed.

## DISCUSSION

Our case series describes endobronchial instillation of ICG in combination with CT‐guided methylene blue to achieve identification of the intersegmental plane, localisation of the target lesion to facilitate successful robotic AS. Using double localisation increases the accuracy of resection. If a discordance is shown, a wider resection will be considered. This technique is appropriate management of small lesions with a high likelihood of being metachronous tumours. We describe minimal morbidity, acceptable inpatient hospital length of stay (3–8 days), and better preservation of lung function. Successful surgical margins were achieved in all three cases.

Small nodules are often not palpable. It is difficult to confirm that the correct tissue is in the resected specimen, frozen section is similarly problematic. Having the correlational accuracy of CT‐guided methylene blue target lesion injection and a tract to the pleural surface of the lung combined with segmental bronchi ICG delineating the segmental border may increase the accuracy of lung nodule resection.

Retrospective and case‐control studies of lobectomy versus segmentectomy demonstrate comparable results for small (Stage 1A <2 cm or 3 cm) tumours.^16^, ^17^, ^18^, ^19^ Local recurrence after segmentectomy appears affected by segment isolation and resection size, compounding the need for precision surgery with regards to AS.^20^

A recently published systematic review compared patients treated with sublobar resection versus lobectomy. In an ‘intentionally selected’ cohort of patients with peripheral, less than 2 cm parenchymal lesions, sublobar resection had similar outcomes to lobectomy in terms of disease‐free and overall survival.^6^ However, despite this, functional benefits from segmentectomy over lobectomy have not yet been firmly established.^21^ RCTs evaluating robotic segmentectomy versus Video‐Assisted Thoracoscopic Surgery (VATS) lobectomy are lacking.

Andolfi et al. published on the various methods of identification of the intersegmental plane during thoracoscopic segmentectomy.^13^ The inflation‐deflation method was widely adopted for decades. Obscuration of the surgical view and incorrect demarcation of the target area by aeration of segments in neighbouring ventilated lung, particularly problematic in emphysematous lobes, has been noted as a negative consequence of this approach.^15^ Endobronchial instillation of indocyanine green (ICG) is highlighted as a technique that does not require lung inflation and therefore avoids these inherent problems associated with collateral ventilation and a more limited, narrow working space for VATS.^13^

Several thoracic surgeons have adopted intravenous injection of ICG via the segmental pulmonary artery. A retrospective cohort study published in 2019 looked at data from a single surgeon at a single facility (*n* = 245). A combination of ICG instilled endobronchially and intravenous ICG injection via injection in the segmental pulmonary artery was performed. In addition to using this method, lesion localization was facilitated by electromagnetic navigational bronchoscopy (ENB) in 38% of cases. Geraci et al. quote 80/93 (86%) patients were found to have the lesion accurately identified using their method. In their cohort, segmentectomy was largely confined to lesions <2 cm, N0/N1 disease and resections that were deemed to be likely to achieve satisfactory surgical margins. Failure of localisation was caused by the inaccurate injection of ICG, failure of the navigational bronchoscopy software or pleural perforation of the dye.^22^

Obtaining the optimal ICG dose for endobronchial injection could be key to reducing the failure rate of this technique. Anayama et al. have estimated that the optimal volume of ICG is 8.91% of the targeted segment volume.^23^

Moving forward, techniques to identify the intersegmental plane are likely to be important in the success of AS as a surgical approach. We believe that endobronchial instillation of ICG and CT guided methylene blue injection for lesion localisation show promise as a technique to complement parenchymal sparing thoracic surgery.

## AUTHOR CONTRIBUTIONS

**Paul Lilburn**: Joint first author. Directly involved in the conception of the manuscript, performed the literature review and critically appraised the articles selected; drafted the manuscript and revised the manuscript for critically important intellectual content. Directly involved in the final approval of the version to be published. **Jonathan Kwan**: Joint first author. Directly involved with the production of the manuscript and detailing the three cases described; drafted the work and revised the manuscript in conjunction with other co‐authors. Directly involved with the final approval of the version to be published. **Jonathan Williamson**: Involved in revising the manuscript for critically important intellectual content. Directly involved in the final approval of the version to be published. **Mohammed Azari** and **Michael Wilson**: Surgeon involved in the selected cases. Involved in revising the manuscript for critically important intellectual content. Directly involved in the final approval of the version to be published. **Alvin Ing**: Bronchoscopist involved in the selected cases. Involved in revising the manuscript for critically important intellectual content. Directly involved in the final approval of the version to be published. **T. Saghaie**: Lead author. Bronchoscopist involved in the selected cases. Directly involved in the conception of the manuscript drafting the work or revising it critically for important intellectual content; and (iii) final approval of the version to be published.

## CONFLICT OF INTEREST STATEMENT

All authors have no conflicts of interest to declare.

## ETHICS STATEMENT

The authors declare that appropriate written informed consent was obtained for the publication of this manuscript and accompanying images.

## Data Availability

The data that support the findings of this study are available from the corresponding author upon reasonable request.

## References

[1] NCI . The surveillance, epidemiology, and end results (SEER). Bethesda, MD: National Institutes of Health; 2021. https://seer.cancer.gov/

[2] Australia AGC . National Cancer Control Indicators: Australia Government Cancer Australia. 2019 https://ncci.canceraustralia.gov.au/diagnosis/cancer-incidence/cancer-incidence

[3] de Koning HJ , van der Aalst CM , de Jong PA , Scholten ET , Nackaerts K , Heuvelmans MA , et al. Reduced lung‐cancer mortality with volume CT screening in a randomized trial. N Engl J Med. 2020;382(6):503–13. 31995683 10.1056/NEJMoa1911793

[4] Team NLSTR . Reduced lung‐cancer mortality with low‐dose computed tomographic screening. N Engl J Med. 2011;365(5):395–409. 21714641 10.1056/NEJMoa1102873 PMC4356534

[5] Ginsberg RJ , Rubinstein LV , Group LCS . Randomized trial of lobectomy versus limited resection for T1 N0 non‐small cell lung cancer. Ann Thorac Surg. 1995;60(3):615–23. 7677489 10.1016/0003-4975(95)00537-u

[6] Cao C , Chandrakumar D , Gupta S , Yan TD , Tian DH . Could less be more?—a systematic review and meta‐analysis of sublobar resections versus lobectomy for non‐small cell lung cancer according to patient selection. Lung Cancer. 2015;89(2):121–32. 26033208 10.1016/j.lungcan.2015.05.010

[7] Hou B , Deng X‐F , Zhou D , Liu Q‐X , Dai J‐G . Segmentectomy versus wedge resection for the treatment of high‐risk operable patients with stage I non‐small cell lung cancer: a meta‐analysis. Ther Adv Respir Dis. 2016;10(5):435–43. 27585599 10.1177/1753465816667121 PMC5933623

[8] Xue W , Duan G , Zhang X , Zhang H , Zhao Q , Xin Z . Meta‐analysis of segmentectomy versus wedge resection in stage IA non‐small‐cell lung cancer. Onco Targets Ther. 2018;11:3369–75. 29922075 10.2147/OTT.S161367 PMC5995300

[9] Zhang H , Liu C , Tan Z , Zhang T . Segmentectomy versus wedge resection for stage I non–small cell lung cancer: a meta‐analysis. J Surg Res. 2019;243:371–9. 31277014 10.1016/j.jss.2019.05.058

[10] Altorki NK , Kamel MK , Narula N , Ghaly G , Nasar A , Rahouma M , et al. Anatomical segmentectomy and wedge resections are associated with comparable outcomes for patients with small cT1N0 non–small cell lung cancer. J Thorac Oncol. 2016;11(11):1984–92. 27496651 10.1016/j.jtho.2016.06.031

[11] Kent M , Landreneau R , Mandrekar S , Hillman S , Nichols F , Jones D , et al. Segmentectomy versus wedge resection for non‐small cell lung cancer in high‐risk operable patients. Ann Thorac Surg. 2013;96(5):1747–55. 23998400 10.1016/j.athoracsur.2013.05.104

[12] Nomori H , Yamazaki I , Machida Y , Otsuki A , Cong Y , Sugimura H , et al. Lobectomy versus segmentectomy: a propensity score‐matched comparison of postoperative complications, pulmonary function and prognosis. Interact Cardiovasc Thorac Surg. 2022;34(1):57–65. 34999814 10.1093/icvts/ivab212 PMC8743134

[13] Andolfi M , Potenza R , Seguin‐Givelet A , Gossot D . Identification of the intersegmental plane during thoracoscopic segmentectomy: state of the art. Interact Cardiovasc Thorac Surg. 2020;30(3):329–36. 31773135 10.1093/icvts/ivz278

[14] Gossot D , Seguin‐Givelet A . The intersegmental plane: an emerging concern for the thoracoscopic surgeon. Video Assist Thorac Surg. 2017;2:34.

[15] Quan YH , Oh CH , Jung D , Lim J‐Y , Choi BH , Rho J , et al. Evaluation of intraoperative near‐infrared fluorescence visualization of the lung tumor margin with indocyanine green inhalation. JAMA Surg. 2020;155(8):732–40. 32579150 10.1001/jamasurg.2020.1314 PMC7439110

[16] Koike T , Yamato Y , Yoshiya K , Shimoyama T , Suzuki R . Intentional limited pulmonary resection for peripheral T1 N0 M0 small‐sized lung cancer. J Thorac Cardiovasc Surg. 2003;125(4):924–8. 12698157 10.1067/mtc.2003.156

[17] Okada M , Nishio W , Sakamoto T , Uchino K , Yuki T , Nakagawa A , et al. Effect of tumor size on prognosis in patients with non–small cell lung cancer: the role of segmentectomy as a type of lesser resection. J Thorac Cardiovasc Surg. 2005;129(1):87–93. 15632829 10.1016/j.jtcvs.2004.04.030

[18] Okumura M , Goto M , Ideguchi K , Tamura M , Sasaki H , Tanaka H , et al. Factors associated with outcome of segmentectomy for non‐small cell lung cancer: long‐term follow‐up study at a single institution in Japan. Lung Cancer. 2007;58(2):231–7. 17673328 10.1016/j.lungcan.2007.06.014

[19] Warren WH , Faber LP . Segmentectomy versus lobectomy in patients with stage I pulmonary carcinoma: five‐year survival and patterns of intrathoracic recurrence. J Thorac Cardiovasc Surg. 1994;107(4):1087–94. 8159031

[20] Okada M , Yoshikawa K , Hatta T , Tsubota N . Is segmentectomy with lymph node assessment an alternative to lobectomy for non–small cell lung cancer of 2 cm or smaller? Ann Thorac Surg. 2001;71(3):956–60. 11269480 10.1016/s0003-4975(00)02223-2

[21] Charloux A , Quoix E . Lung segmentectomy: does it offer a real functional benefit over lobectomy? Eur Respir Rev. 2017;26(146):170079. 29070582 10.1183/16000617.0079-2017 PMC9488724

[22] Geraci TC , Ferrari‐Light D , Kent A , Michaud G , Zervos M , Pass HI , et al. Technique, outcomes with navigational bronchoscopy using indocyanine green for robotic segmentectomy. Ann Thorac Surg. 2019;108(2):363–9. 30980818 10.1016/j.athoracsur.2019.03.032

[23] Anayama T , Hirohashi K , Miyazaki R , Okada H , Yamamoto M , Orihashi K . Fluorescence visualization of the intersegmental plane by bronchoscopic instillation of indocyanine green into the targeted segmental bronchus: determination of the optimal settings. J Int Med Res. 2021;49(2):300060521990202. 33567948 10.1177/0300060521990202 PMC7883170

